# Exacerbation of Pre-existing Hypothyroidism Following Enarodustat Administration in a Patient on Peritoneal Dialysis: A Case Report

**DOI:** 10.7759/cureus.116337

**Published:** 2026-09-16

**Authors:** Kiho Yanagisawa, Yukihiro Wada, Shun Sakurabayashi, Hiroyuki Okawa, Genichi Yanagisawa, Mayuko Hanyuda, Kensuke Terakawa, Yuki Toyoda, Rina Kamiya, Ryota Uchitsubo, Sayumi Kawamura, Tomomi Motohashi, Hiroshi Tominaga, Kazuhiro Takeuchi, Yasuo Takeuchi

**Affiliations:** 1 Department of Nephrology, Kitasato University School of Medicine, Sagamihara, JPN

**Keywords:** enarodustat, hyperparathyroidism, hypothyroidism, peritoneal dialysis (pd), renal anemia

## Abstract

Hypoxia-inducible factor prolyl hydroxylase (HIF-PH) inhibitors are widely used to treat renal anemia in patients with chronic kidney disease (CKD). While roxadustat, an HIF-PH inhibitor, is known to induce central hypothyroidism via thyroid hormone receptor-beta agonism, thyroid dysfunction associated with other HIF-PH inhibitors, such as enarodustat, remains unrecognized. We report the case of a 32-year-old male on peritoneal dialysis (PD) with underlying subclinical mild primary hypothyroidism and erythropoiesis-stimulating agent (ESA)-resistant renal anemia secondary to primary hyperparathyroidism. Following a switch from ESAs to enarodustat and its subsequent dose escalation (up to 6 mg/day), the patient experienced severe exacerbation of hypothyroidism characterized by markedly elevated thyroid-stimulating hormone (TSH: 139 µIU/mL) and decreased free thyroxine (FT4: 0.42 ng/dL), culminating in acute decompensated heart failure that required emergency admission and temporary hemodialysis. Initial emergency management with blood purification therapy successfully stabilized his respiratory status, whereas treatment with levothyroxine alone had failed to control his TSH levels. Discontinuation of enarodustat, combined with levothyroxine titration, led to a gradual restoration of thyroid function and resolution of systemic symptoms, allowing the successful resumption of PD. Unlike the central hypothyroidism caused by roxadustat, enarodustat may have contributed to the exacerbation of pre-existing hypothyroidism, suggesting a distinct pathophysiological mechanism, such as impaired thyroid hormone synthesis or secretion. To our knowledge, this is the first report demonstrating that a non-roxadustat HIF-PH inhibitor, enarodustat, could cause severe exacerbation of hypothyroidism in a CKD patient. These findings highlight the critical need to closely monitor thyroid function when prescribing any HIF-PH inhibitor, particularly in patients with pre-existing thyroid disorders.

## Introduction

Renal anemia is a major complication of end-stage renal disease (ESRD) [1,2]. Hypoxia-inducible factor prolyl hydroxylase (HIF-PH) inhibitors have emerged as effective oral alternatives to conventional erythropoiesis-stimulating agents (ESAs) by stimulating endogenous erythropoietin production and improving iron metabolism [2-4]. Currently, several HIF-PH inhibitors, including roxadustat, daprodustat, vadadustat, enarodustat, and molidustat, are approved in clinical practice [2]. Furthermore, growing evidence suggests that HIF-PH inhibitors are particularly beneficial for managing ESA-resistant anemia [5].

Meanwhile, accumulating evidence indicates that roxadustat can induce central hypothyroidism [6-8]. According to previous reports, roxadustat possesses a molecular structure similar to triiodothyronine (T3) and acts as an agonist for thyroid hormone receptor-beta (THR-β) in the hypothalamus and pituitary gland, suppressing thyroid-stimulating hormone (TSH) secretion via negative feedback mechanisms [9]. Consequently, roxadustat-induced thyroid dysfunction typically presents with suppressed or normal TSH levels alongside reduced free thyroxine (FT4) levels [6,8,10-12]. Meanwhile, adverse effects on thyroid function caused by non-roxadustat HIF-PH inhibitors, particularly enarodustat, have not been clearly characterized.

Here, we report a rare case of peritoneal dialysis (PD) with underlying primary hypothyroidism who experienced a severe exacerbation of hypothyroidism, characterized by markedly elevated TSH and low FT4 levels following enarodustat therapy. We highlight the potential for enarodustat-related hypothyroidism and suggest a novel mechanism of HIF-PH inhibitor-induced thyroid toxicity.

## Case presentation

A 32-year-old male with ESRD due to nephrosclerosis had been maintained on continuous ambulatory peritoneal dialysis (CAPD) for two years (three 2.0 L exchanges daily using a 1.5% glucose solution). His past medical history was notable for resection of neuroblastoma at six months of age and subarachnoid hemorrhage at 26 years of age. He had no remarkable family history of hypertension or endocrine disorders. At CAPD initiation, baseline laboratory parameters showed hemoglobin 12.3 g/dL, transferrin saturation (TSAT) 16.2%, and ferritin 127 ng/mL. He had persistent hyperparathyroidism with an intact parathyroid hormone (iPTH) level of 416 pg/mL associated with primary hyperparathyroidism (PHPT) due to a single enlarged right lower parathyroid gland with internal blood flow (Figure 1), which was refractory to the evocalcet treatment. In addition, thyroid function data at CAPD initiation indicated mild primary hypothyroidism, with a TSH of 5.80 µIU/mL and an FT4 of 0.82 ng/dL, along with negative anti-thyroid peroxidase (anti-TPO) antibodies (<10 IU/mL). Anti-thyroglobulin antibody (TgAb) was 15 IU/mL, and thyroglobulin was 129.0 ng/mL. Neck ultrasonography revealed no remarkable lesions in the thyroid gland (Figure 2). Because the biochemical abnormality was mild and the patient had no typical thyroid-related symptoms, levothyroxine was not initiated, and thyroid function was closely monitored. Thereafter, the patient developed ESA-resistant anemia, which was likely exacerbated by underlying PHPT. Despite treatment with continuous erythropoietin receptor activator (CERA), his hemoglobin level dropped to 8.4 g/dL. Consequently, seven months before admission, CERA was switched to enarodustat at 2 mg/day. Because of an insufficient therapeutic response, the dose of enarodustat was increased to 4 mg/day and subsequently to 6 mg/day two months before admission. Following the dose escalation of enarodustat, his thyroid function markedly deteriorated: TSH rose to 45.8 µIU/mL, with a free triiodothyronine (FT3) level of 2.1 pg/mL and an FT4 level of 0.73 ng/dL. This thyroid dysfunction prompted the immediate initiation of levothyroxine sodium at 25 µg/day. However, his hypothyroidism rapidly worsened; 12 days before admission, his TSH further elevated to 139 µIU/mL, accompanied by decreases in FT3 to 1.94 pg/mL and FT4 to 0.42 ng/dL. Consequently, the patient required emergency admission due to severe dyspnea.

**Figure 1:**
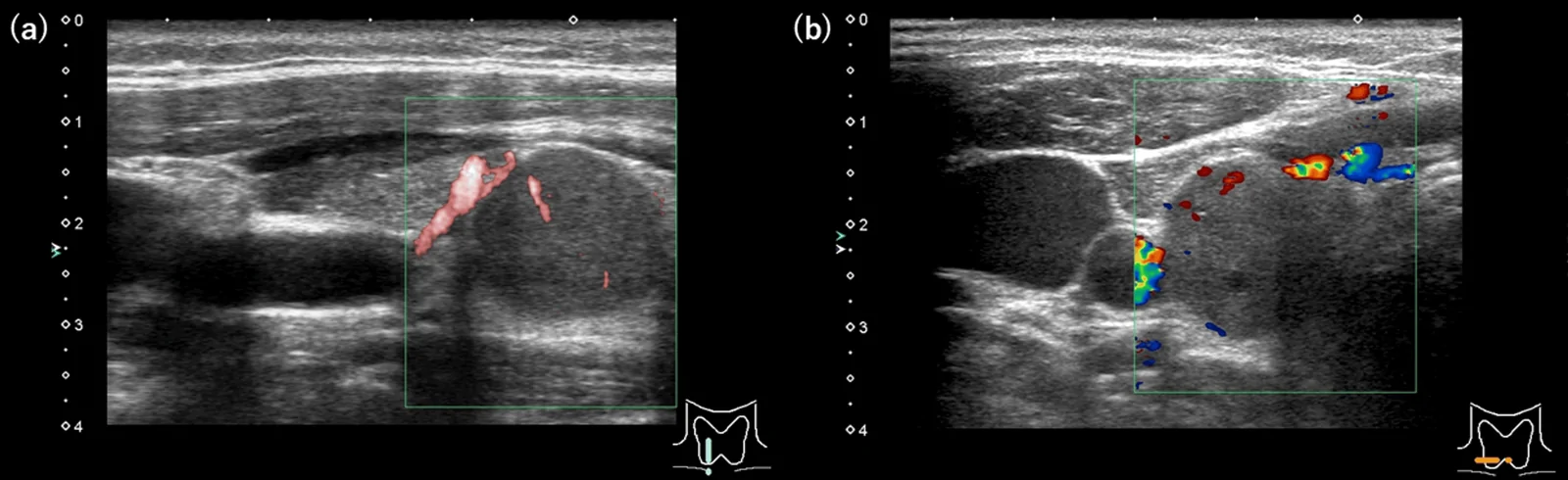
Neck ultrasonography showing a single enlarged parathyroid gland. (a) Longitudinal and (b) transverse ultrasound images revealing a single, hypoechoic enlarged right lower parathyroid gland with internal blood flow.

**Figure 2:**
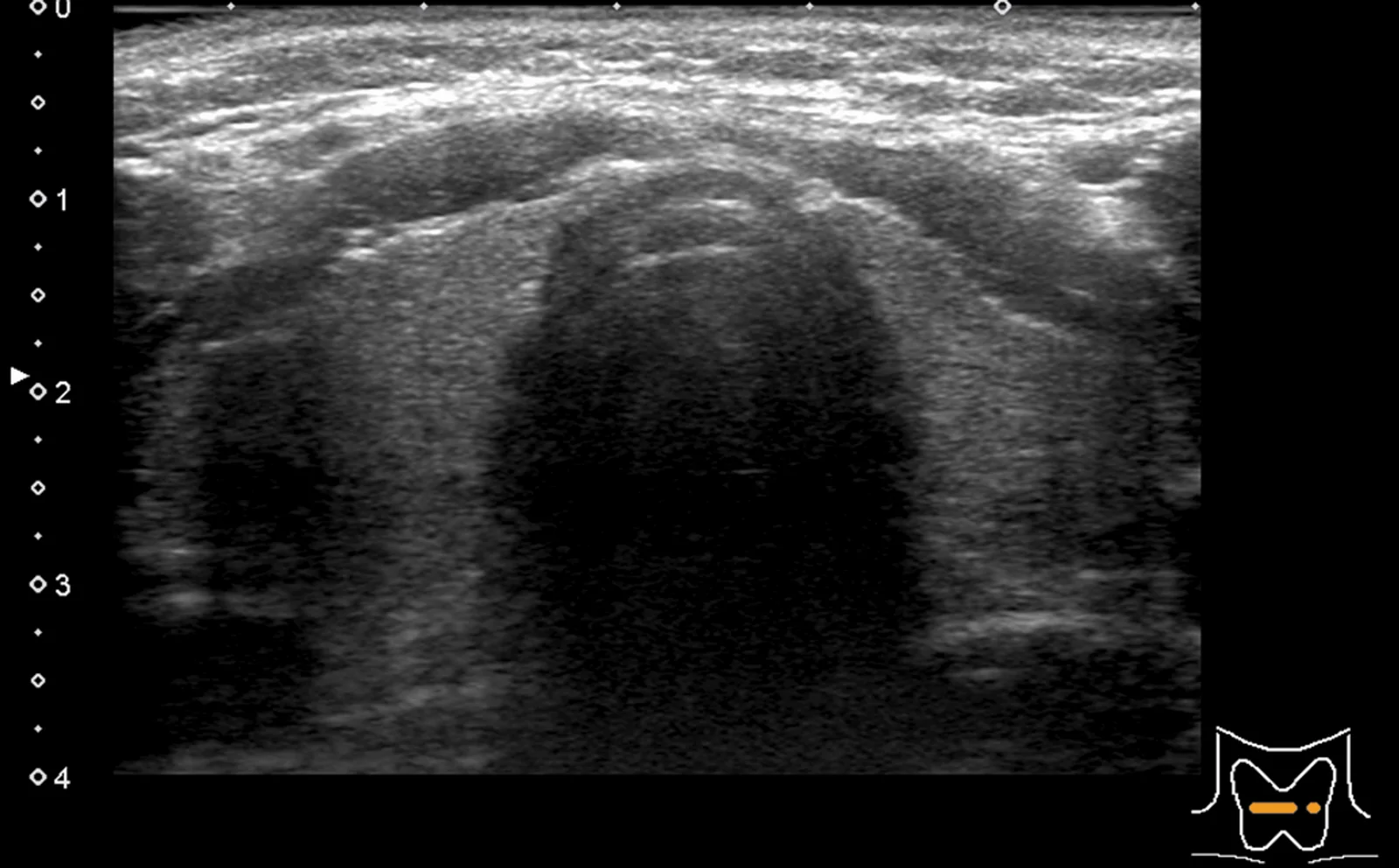
Anterior neck ultrasonography of the thyroid gland. Transverse ultrasound image demonstrating a normal thyroid parenchyma with no focal or space-occupying lesions.

At emergency admission, physical examination revealed severe respiratory distress (respiratory rate: 35 breaths/minute, SpO_2_: 98% on a 10 L/minute mask), severe hypertension (217/148 mmHg), tachycardia (142 beats/minute), coarse crackles in both lower lung fields, abdominal distension, and bilateral pretibial edema without impairment of consciousness and hypothermia. Laboratory findings at admission are summarized in Table 1. Hematological evaluation demonstrated normocytic normochromic anemia (hemoglobin: 10.5 g/dL, mean corpuscular volume: 95.4 fL, mean corpuscular hemoglobin concentration: 31.2%) with an inadequate reticulocyte response (0.3%). Iron profile revealed decreased serum iron (20 µg/dL) and TSAT (6.2%), along with a mildly decreased total iron-binding capacity (323 µg/dL) and low-normal ferritin level (98 ng/mL). Blood chemistry showed severe renal dysfunction (blood urea nitrogen: 95.5 mg/dL, creatinine: 19.4 mg/dL), elevated creatine kinase (CK: 569 IU/L), and severe hyperphosphatemia (phosphorus: 8.7 mg/dL), whereas other serum electrolyte levels remained within normal limits. Immunological testing demonstrated a mild elevation of C-reactive protein (1.47 mg/dL) with negative autoantibodies. Markedly elevated N-terminal prohormone of brain natriuretic peptide (15,436 pg/mL) indicated severe volume overload. Endocrine and bone metabolic evaluation confirmed severe primary hypothyroidism (TSH: 103 µIU/mL, FT3: 0.5 pg/mL, FT4: 0.47 ng/dL) with negative thyroid-specific autoantibodies (anti-TPO and anti-Tg Abs), as well as persistent hyperparathyroidism accompanied by markedly elevated bone turnover markers (iPTH: 356 pg/mL, osteocalcin: 261 ng/mL). Regarding the images on admission, chest radiography demonstrated marked pulmonary congestion and cardiomegaly, whereas electrocardiography showed no ischemic changes. Neck ultrasonography reconfirmed a single enlarged parathyroid gland located at the lower pole of the right thyroid lobe, with no visible discrete lesions within the thyroid gland itself.

**Table 1:** Laboratory findings on the second admission. WBC = white blood cell; RBC = red blood cell; MCV = mean corpuscular volume; MCHC = mean corpuscular hemoglobin concentration; TP = total protein; GOT = aspartate aminotransferase; GPT = alanine aminotransferase; LDH = lactate dehydrogenase; CK = creatine kinase; BS = blood sugar; HbA1c = hemoglobin A1c; TG = triglyceride: LDL = low-density lipoprotein cholesterol; HDL = high-density lipoprotein cholesterol; BUN = blood urea nitrogen; UA = uric acid; eGFR = estimated glomerular filtration rate; Na = sodium; K = potassium; Cl = chloride; Ca = calcium; P = phosphorus; Fe = serum iron; TIBC = total iron-binding capacity; TSAT = transferrin saturation; TSH = thyroid-stimulating hormone; FT3 = free triiodothyronine; FT4 = free thyroxine; Anti-TPO Ab = anti-thyroid peroxidase antibody; Anti-Tg Ab = anti-thyroglobulin antibody; iPTH = intact parathyroid hormone; NT-proBNP = N-terminal prohormone of brain natriuretic peptide; CRP = C-reactive protein; IgG = immunoglobulin G; IgA = immunoglobulin A; IgM = immunoglobulin M; C3 = component 3; C4 = component 4; CH50 = total hemolytic complement activity; ANA = antinuclear antibody

Parameter	Unit	Value	Reference range
WBC count	µL^-1^	14,300	3,300–8,600
RBC count	µL^-1^	3.48 × 10^6^	4.10–5.30
Hemoglobin	g/dL	10.5	13.7–16.8
MCV	fL	95.4	80–100
MCHC	%	31.2	30.2–35.1
Reticulocyte	%	0.3	0.2–2.7
Platelet count	10^4^/µL	23.0	15.8–34.8
TP	g/dL	5.9	6.0–8.0
Albumin	g/dL	3.4	4.1–5.1
Total bilirubin	mg/dL	0.4	0.2–1.2
GOT	IU/L	13	10–40
GPT	IU/L	9	10–40
LDH	IU/L	413	140–280
CK	IU/L	569	41–153
Amylase	IU/L	136	44–132
BS	mg/dL	116	73–109
HbA1c	%	4.7	4.6–6.2
TG	mg/dL	164	30–149
LDL	mg/dL	117	65–139
HDL	mg/dL	35	40–103
BUN	mg/dL	95.5	8.0–20.0
Creatinine	mg/dL	19.4	0.6–1.0
eGFR	mL/minute	2.4	>60
UA	mg/dL	8.9	2.6–7.0
Na	mEq/L	139	138–145
K	mEq/L	4.5	3.5–5.0
Cl	mEq/L	99	101–108
Ca	mg/dL	8.6	8.8–10.0
P	mg/dL	8.7	2.5–4.7
Fe	μg/dL	20	60–200
TIBC	μg/dL	323	253–365
TSAT	%	6.2	20–50
TSH	μlU/mL	103	0.4–4.0
FT3	pg/mL	0.5	2.0–4.4
FT4	ng/dL	0.47	0.8–1.8
Thyroglobulin	ng/mL	129.0	<33.7
Anti-TPO Ab	IU/mL	<3.3	<3.3
Anti-Tg Ab	IU/mL	15	<28
iPTH	pg/mL	356	10–65
Osteocalcin	ng/mL	261	3–30
NT-proBNP	pg/mL	15,436	<125
CRP	mg/dL	1.47	0.0–0.3
Ferritin	ng/mL	98	15–150
IgG	mg/dL	568	870–1,700
IgA	mg/dL	102	110–410
IgM	mg/dL	29	52–270
C3	mg/dL	49	80–140
C4	mg/dL	16	15–45
CH50	U/mL	54	30–45
ANA	Titer	<40	<40

The clinical course after admission is shown in Figure 3. Upon admission (day zero), continuous hemodiafiltration (CHDF) was initiated to manage severe uremia, heart failure, and volume overload. Following clinical stabilization with CHDF, the patient was successfully transitioned to CAPD (1.5% PD solution, 2 L, four exchanges/day). Regarding anemia management, enarodustat was immediately discontinued upon admission, and CERA (150 µg) was reinitiated alongside adequate iron supplementation. Under this regimen, the patient’s hemoglobin levels were stably maintained between 8.0 and 10.0 g/dL during hospitalization. For severe hypothyroidism, oral levothyroxine was progressively titrated from 25 µg/day to 50 µg/day, 75 µg/day, and eventually 87.5 µg/day. Following enarodustat cessation and levothyroxine titration, serum TSH levels steadily declined (48 µIU/mL on hospital day 48, FT4: 0.64 ng/dL), accompanied by clinical recovery. The patient was discharged on hospital day 48. At seven months post-discharge, under maintenance levothyroxine therapy (87.5 µg/day), his thyroid function had normalized (TSH: 2.57 µIU/mL, FT4: 1.12 ng/dL). Additionally, the patient has successfully continued CAPD, with hemoglobin levels adequately maintained at 10.0-12.0 g/dL on CERA alone, without requiring the resumption of enarodustat or other HIF-PH inhibitors.

**Figure 3:**
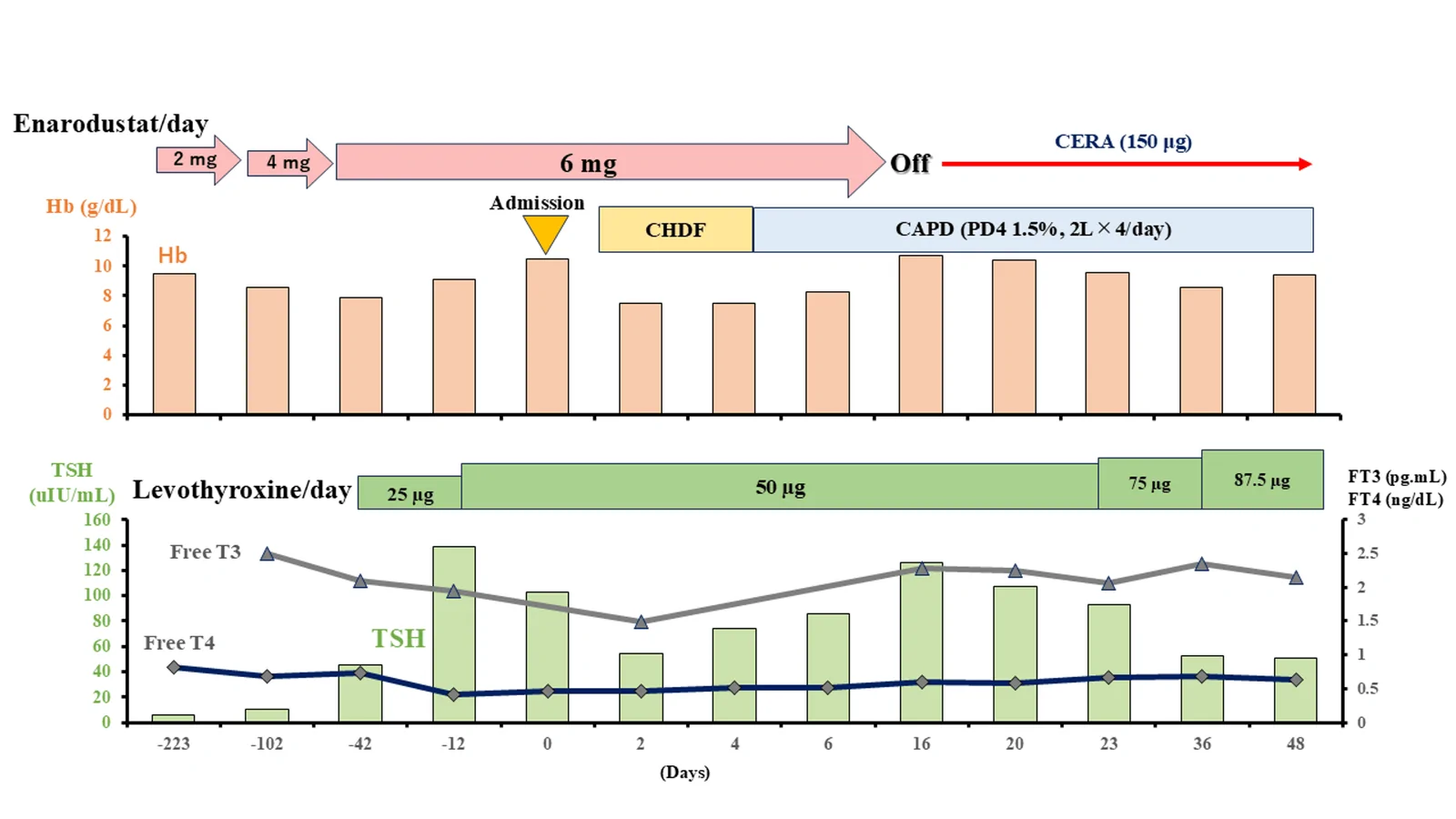
Clinical course in the present case. The graph shows changes in hemoglobin levels, thyroid function parameters, and therapeutic interventions before and after admission. Hb = hemoglobin; CHDF = continuous hemodiafiltration; CAPD = continuous ambulatory peritoneal dialysis; CERA = continuous erythropoietin receptor activator; TSH = thyroid-stimulating hormone; FT3 = free triiodothyronine; FT4 = free thyroxine

In short, the clinical timeline was summarized as follows: (i) Baseline subclinical primary hypothyroidism was documented at CAPD initiation. (ii) Enarodustat was started seven months before admission and escalated to 6 mg/day two months before admission. (iii) Thyroid function deteriorated following the dose escalation of enarodustat. (iv) Severe hypothyroidism and volume overload prompted emergency admission. (v) Enarodustat was discontinued upon admission. (vi) Thyroid function subsequently normalized with levothyroxine replacement.

## Discussion

The present case provides critical insights regarding the management of ESA-resistant renal anemia and the potential adverse effects of HIF-PH inhibitors in patients undergoing PD. In retrospect, the primary clinical point in this patient was the management of ESA-resistant anemia. Although therapy-resistant PHPT appeared to serve as the chief cause of anemia, inadequate iron supplementation and underdialysis, that is attributable to insufficient solute clearance on PD in the setting of his deteriorating systemic condition, partially contributed to the persistent anemia. Given the lack of response to medical therapy for PHPT and the presence of severe ESA resistance in a young adult, parathyroidectomy should have been strongly proposed and pursued with thoroughly informed patient consent. Furthermore, when switching from ESAs to enarodustat, iron agents should have been aggressively supplemented to achieve higher target TSAT and ferritin levels. Generally, initiating or escalating HIF-PH inhibitors under iron-deficient conditions carries an elevated risk of thromboembolic events [13,14]; thereby, the initiation of enarodustat should have been delayed until sufficient iron replenishment was achieved.

Despite the patient’s complex clinical condition, the marked exacerbation of thyroid dysfunction following enarodustat initiation and dose escalation, with no evidence of autoimmune thyroiditis, suggests a causal relationship, indicating that enarodustat might be a plausible contributor to the thyroid deterioration. Meanwhile, unlike the typical presentation of severe hypothyroidism with hypotension and bradycardia, this case exhibited hypertension and tachycardia. We speculate that this atypical feature resulted from persistent fluid overload due to inadequate PD management and a pre-existing history of hypertension, leading to congestive heart failure. Furthermore, as another notable point, the recovery of thyroid function remained prolonged following the prompt discontinuation of enarodustat, resolution of uremia and fluid overload via CHDF, and optimization of levothyroxine therapy. Although initial severe uremia and volume overload at admission could theoretically cause non-thyroidal illness syndrome (NTIS) [15,16], intensive treatment with CHDF successfully cleared these systemic factors (Figure 3). Therefore, NTIS alone cannot fully explain the clinical course in this case. First, while NTIS typically presents with normal or only mildly altered TSH levels [16], our patient exhibited a markedly elevated TSH. Second, despite the resolution of systemic insults, severe hypothyroidism persisted, likely because enarodustat was inadvertently continued during early admission. These findings indicate that enarodustat might exert a sustained pharmacological interference with thyroid function that extends well beyond the resolution of acute illnesses. Moreover, in the clinical setting, subclinical or mild hypothyroidism is occasionally encountered in patients with ESRD; however, it typically affects older individuals on maintenance hemodialysis and is usually manageable with low-to-moderate doses of levothyroxine [17,18]. In contrast, our young patient developed severe hypothyroidism that required high-dose levothyroxine supplementation. Therefore, the mild thyroid dysfunction typically observed in ESRD patients cannot account for the refractory and severe clinical course observed in our patient.

To our knowledge, there have been no reports regarding enarodustat-related hypothyroidism, in contrast to roxadustat-induced thyroid dysfunction [11]. Unlike roxadustat, which typically induces central hypothyroidism via THR-β activation [9], enarodustat may affect thyroid function through a distinct mechanism. When considering the influence of HIF-PH inhibitors on thyroid cellular mechanisms, several reports imply that activation of the HIF pathway could directly suppress key molecules involved in thyroid hormone synthesis [19-21]. Specifically, HIF activation has been reported to downregulate the gene expression of the sodium-iodide symporter, which mediates follicular iodide uptake [19,20]. In other words, HIF activation induced by HIF-PH inhibitors may impair the intracellular uptake of iodide, an essential substrate required for thyroid hormone synthesis. Furthermore, in our patient, TSH failed to normalize with continued enarodustat despite levothyroxine titration and remained slow to recover after discontinuation. This clinical course, together with its pharmacological characteristics, raises the possibility that enarodustat exerts a suppressive effect on thyroid hormone synthesis, secretion, or signal transduction as a potential mechanism. Additionally, it is suggested that enarodustat-induced thyroid suppression may manifest in a severe and dose-dependent manner, where simple drug withdrawal may not lead to prompt spontaneous recovery.

However, several limitations of this case report should be noted. First, because this is a single-case report and thyroid dysfunction persisted after drug discontinuation, a definitive causal relationship cannot be firmly established. Second, this severe exacerbation occurred in a patient with pre-existing hypothyroidism; thus, such an effect is unlikely to be observed in individuals with normal baseline thyroid function. Finally, it remains uncertain whether this phenomenon is driven by a class effect of HIF-PH inhibitors or drug-specific toxicity unique to enarodustat. Further studies are warranted to clarify these mechanisms and the interaction between enarodustat and thyroid hormone metabolism.

## Conclusions

Enarodustat might cause severe exacerbation of pre-existing primary hypothyroidism, distinct from the central hypothyroidism associated with roxadustat. Clinicians should exercise caution when prescribing enarodustat to patients with underlying subclinical or overt hypothyroidism, and routine monitoring of TSH and FT4 is recommended during the initiation or dose titration of HIF-PH inhibitors.
